# Supplementary material for: Stalled response near thermal equilibrium in periodically driven systems
Source: Nat Commun. 2024 Jan 4;15:294. doi: 10.1038/s41467-023-44487-2 (PMC10766978; doi:10.1038/s41467-023-44487-2)
Supplement: Supplementary file 1 — Supplementary Information [file 41467_2023_44487_MOESM1_ESM.pdf]

Supplementary Information for
"Stalled response near thermal equilibrium in periodically driven systems"

Lennart Dabelow[1,2] and Peter Reimann[3]

[1] *RIKEN Center for Emergent Matter Science (CEMS), Wako, Saitama 351-0198, Japan*
[2] *School of Mathematical Sciences, Queen Mary University of London, London E1 4NS, UK*
[3] *Faculty of Physics, Bielefeld University, 33615 Bielefeld, Germany*

(Dated: November 28, 2023)

Supplementary Note 1: Response characteristics

1.1. *Response and heating time scales*

Generically, a many-body system is expected to absorb energy and thus heat up under periodic driving [1–9]. As explained in the main paper, a key prerequisite to observe stalled response is that these heating effects are sufficiently suppressed such that the dynamics remains practically confined to the initially occupied (microcanonical) energy shell for the relevant response and stalling time scales.

As a first general guess, the characteristic time scale t_{resp} of the observable response for a system away from equilibrium will often be on the order of the characteristic driving time scale T and thus decrease as $t_{\text{resp}} = \mathrm{O}(T)$ for small T. Within our theory (cf. Eq. (9) in the main paper), the response is encoded in $|\gamma_t(t)|^2$. Hence, t_{resp} is the typical time scale of the solutions of Eq. (10) in the main paper, an example of which is shown in Supplementary Fig. 1. For concreteness, we take t_{resp} to be the time at which the first minimum of $|\gamma_t(t)|^2$ is assumed (see also Supplementary Fig. 1) and illustrate its dependence on T in Supplementary Fig. 2a, where we indeed observe a linear relationship in the small-T regime.

For a system that is initially out of equilibrium, *stalling* of the observable response and relaxation to the prethermal plateau are then predicted to occur on the time scale t_{stall} on which the dynamics $\langle A \rangle_{\rho_0(t)}$ of the associated unperturbed system H_0 thermalizes. Since no assumptions about the unperturbed system are made, t_{stall} is largely arbitrary and can vary considerably, similarly to the relaxation times of isolated many-body systems. To observe a noticeable effect of the driving and its subsequent stalling, we need $t_{\text{resp}} < t_{\text{stall}}$ (hence $T \lesssim t_{\text{stall}}$). Moreover, t_{stall} must be considerably smaller than the heating time scale t_{heat}.

Contrary to t_{resp}, this time scale t_{heat} for heating will usually grow as T is decreased. Specifically, approximate laws and rigorous upper bounds for the energy absorption rate per degree of freedom, $\Gamma \sim t_{\text{heat}}^{-1}$, have been established in various lattice systems, for example: for spins or fermions with local interactions in the linear response regime [10] and beyond [11]; for spins with few-body interactions based on truncated Floquet-Magnus expansions [12]; or for hard-core bosons by numerical linked-cluster expansions [6]. All those works demonstrate that $\Gamma \leq \mathrm{e}^{-\mathrm{O}(1/T)}$ asymptotically for small T, opening up a large initial time window where heating effects are insignificant on the typical response and stalling time scales if the driving period is sufficiently small.

Intuitively, one expects that the system can no longer

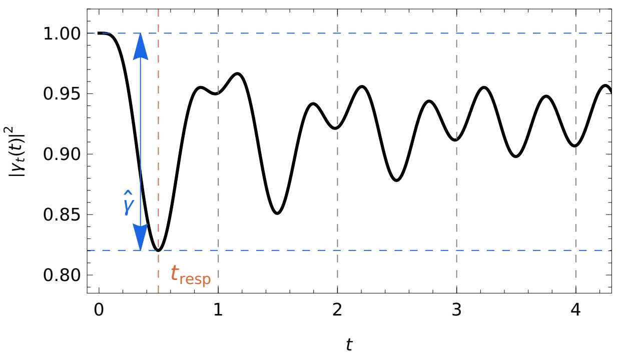

Supplementary Fig. 1. Exemplary squared response function $|\gamma_t(t)|^2$ (cf. Eq. (9) in the main paper), obtained by numerical integration of Eq. (10) from the main paper for an exponential perturbation profile $\tilde{v}(E) = \mathrm{e}^{-|E|}$ and sinusoidal driving $f(t) = \sin(2\pi t)$ (unit period and amplitude). Dashed gray lines: multiples of the driving period $T = 1$. Dashed orange line: response time scale t_{resp}, defined as the location of the first minimum of $|\gamma_t(t)|^2$. Difference between dashed blue lines: response magnitude $\hat{\gamma}$, cf. Supplementary Eq. (1).

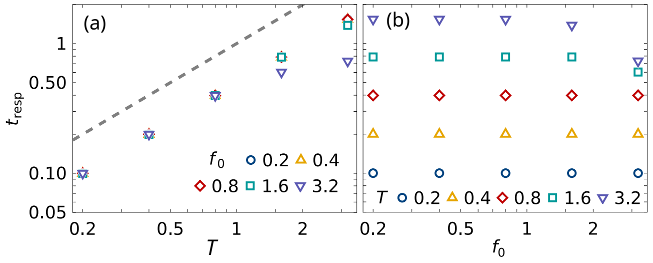

Supplementary Fig. 2. Dependence of the response time scale t_{resp}, defined as the time of the first minimum of $|\gamma_t(t)|^2$, on the driving period T and amplitude f_0 of the sinusoidal driving $f(t) = f_0 \sin(2\pi t/T)$ (cf. Eq. (6) in the main paper). (a) t_{resp} vs. T for various f_0; (b) t_{resp} vs. f_0 for various T. The function $|\gamma_t(t)|^2$ was evaluated by numerically integrating Eq. (10) from the main paper for an exponential perturbation profile $\tilde{v}(E) = \mathrm{e}^{-|E|}$. Dashed line: $t_{\text{resp}} \propto T$ as a guide to the eye.

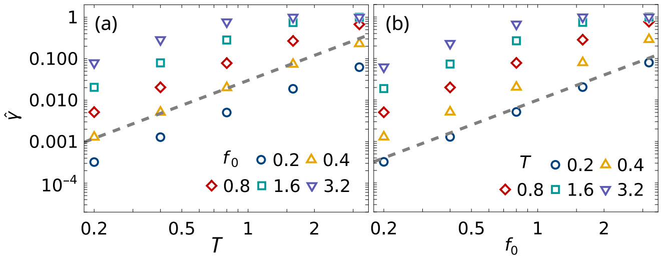

Supplementary Fig. 3. Dependence of the response amplitude $\hat{\gamma}$ from Supplementary Eq. (1) on the driving period T and amplitude f_0. (a) $\hat{\gamma}$ vs. T for various f_0; (b) $\hat{\gamma}$ vs. f_0 for various T. The function $|\gamma_t(t)|^2$ was evaluated by numerically integrating Eq. (10) from the main paper for an exponential perturbation profile $\tilde{v}(E) = \mathrm{e}^{-|E|}$ and sinusoidal driving $f(t) = f_0 \sin(2\pi t/T)$. Dashed lines: $\hat{\gamma} \propto T^2, f_0^2$ as a guide to the eye.

follow the driving for $T \to 0$, i.e., the driving effects average out to zero for asymptotically fast driving. Moreover, the leading order corrections for finite T are expected to be invariant under a sign change of T, i.e., they generically should scale quadratically with T. Within the theory (cf. Eq. (9) in the main paper), we can assess the magnitude of the response as the amplitude of $|\gamma_t(t)|^2$ at the first minimum. Recalling that $\gamma_\tau(0) = 1$, we thus inspect the quantity

$$\hat{\gamma} := 1 - |\gamma_{t_{\mathrm{resp}}}(t_{\mathrm{resp}})|^2 \qquad (1)$$

and find that $\hat{\gamma}$ indeed scales quadratically with T for small values, as shown in Supplementary Fig. 3a. To achieve a noticeable observable response, one should thus increase the amplitude f_0 of the driving if T is decreased. As illustrated in Supplementary Fig. 3b, $\hat{\gamma}$ likewise scales quadratically with f_0 for fixed (sufficiently small) T, whereas the time scale t_{resp} is largely unaffected by such variations of f_0 (cf. Supplementary Fig. 2b). Consequently, a decrease of T can be compensated by a proportional increase of f_0 to maintain a similar magnitude of the observable response; see also Fig. 1 in the main paper for a visualization in a concrete example system.

The heating rate $\Gamma \sim t_{\mathrm{heat}}^{-1}$, in turn, also grows quadratically with f_0 for small amplitudes within the linear response regime according to Refs. [6–8]. However, as mentioned above, the observable response will be very weak if both f_0 and T are small. More precisely, except for finite-size effects, we still expect that the *relative* difference in response between systems near and far from thermal equilibrium will remain significant in this case, but the stalling effect will be less impressive on an absolute scale.

Hence more interesting is the case of stronger driving beyond the linear response regime. Here, general statements about the heating rate Γ are scarce and require more information about the driving operator, but there is evidence that the dependence of Γ on f_0 is often nonmonotonic, such that Γ may decrease again eventually as f_0 becomes larger [7–9]. In any case, the dependence is typically subexponential. As T is decreased, and even if f_0 is increased accordingly, the exponential suppression of heating in $1/T$ will thus eventually dominate. For sufficiently small T and large f_0, we therefore generically expect a regime where heating is insignificant, with a significant response away from equilibrium, but strongly suppressed response in thermal equilibrium.

In conclusion, the parameter regime for stalled response essentially coincides with the one for the theoretically and experimentally well-established phenomenon of Floquet prethermalization [6, 11–17] (see also the discussion in the fifth paragraph of the section "Interpretation and further examples" in the main paper).

1.2. General properties of $\gamma_\tau(t)$

Expanding on the discussion of their time scales and amplitudes in the previous subsection, we collect a few general properties of the solutions $\gamma_\tau(t)$ of the nonlinear integro-differential equation (10) in the main paper. Some of these properties are more easily understood from alternative representations of $\gamma_\tau(t)$, such as Eq. (23) in the main paper, or the Supplementary Eq. (29), defined below in Supplementary Note 6. The equivalence of these representations and Eq. (10) in the main paper will be established in Supplementary Note 7 below. The present section merely serves as a convenient overview.

For all $\tau \in \mathbb{R}$, $\gamma_\tau(t)$ satisfies the initial condition

$$\gamma_\tau(0) = 1 \qquad (2)$$

and is bounded,

$$|\gamma_\tau(t)| \leq 1\,, \qquad (3)$$

see below Supplementary Eq. (55). Furthermore, $u(E, \tau)$ from Supplementary Eq. (27) is real-valued and, for the considered perturbation ensembles as defined in Supplementary Note 5, an even function of E. Since $\gamma_\tau(t)$ is the Fourier transform of $u(E, \tau)$ according to Supplementary Eq. (29), it inherits those properties, i.e.,

$$\gamma_\tau(t) \in \mathbb{R}\,, \quad \gamma_\tau(-t) = \gamma_\tau(t)\,. \qquad (4)$$

Similarly, taking for granted that $u(E, \tau)$ is sufficiently regular, we can conclude that

$$\lim_{t\to\infty} \gamma_\tau(t) = 0 \qquad (5)$$

for any fixed τ. We point out, though, that the long-time behavior of $\gamma_\tau(t)$ is of minor interest for our present purposes because, as discussed in the Methods of the main paper, the truncated Magnus expansion adopted to derive the theoretical prediction from Eq. (9) in the main paper will eventually invalidate it at long times.

As for the short-term behavior, we can immediately infer from Supplementary Eq. (4) (or from the integro-differential equation (10) in the main paper) that

$$\dot{\gamma}_\tau(0) = 0\,. \qquad (6)$$

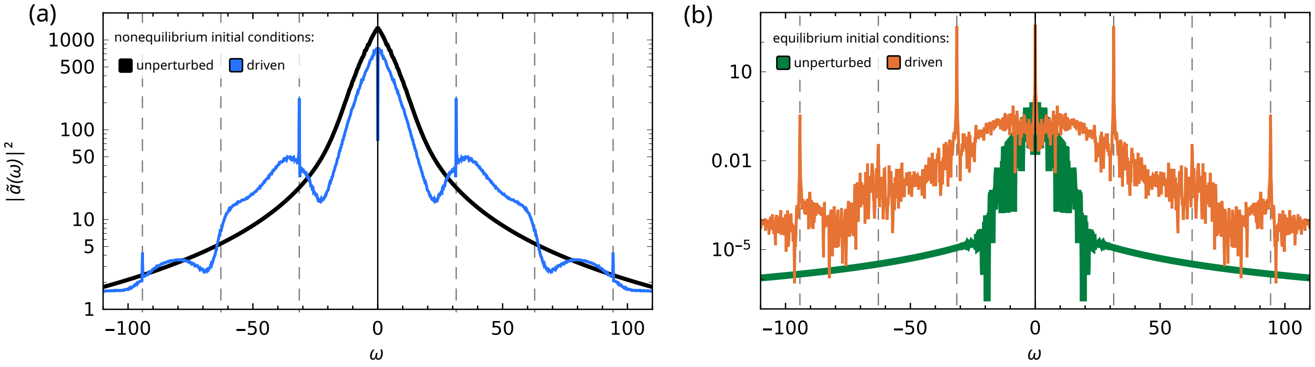

Supplementary Fig. 4. Logarithmically plotted Fourier spectrum $|\tilde{a}(\omega)|^2$ of the nonequilibrium signal $a(t)$ [see Supplementary Eqs. (9)–(10)] for the magnetization correlation $A = \sigma^z_{2,2}\sigma^z_{3,3}$ in the two-dimensional spin lattice from Fig. 1 of the main paper for (a) nonequilibrium and (b) equilibrium initial conditions. Solid lines: discrete Fourier transformation of the corresponding numerical data in Fig. 1 as indicated in the legend. The time series extend up to $t = t_{\max} = 32$ with a step size of $\Delta t = 0.005$, yielding a frequency resolution $\Delta\omega = \frac{\pi}{16} \approx 0.2$ in the range $|\omega| \leq 200\pi \approx 630$. Vertical dashed lines: multiples of the driving frequency $\Omega = 2\pi/T = 10\pi$.

More generally, we can expand $\gamma_\tau(t)$ into a Taylor series around $t = 0$. To this end, it is convenient to introduce the abbreviation $v_\tau(t) := a_\tau v(t) + b_\tau \ddot{v}(t)$, see also Supplementary Eq. (51). Denoting by $\gamma_\tau^{(n)}(t)$ the n-th derivative with respect to t, a straightforward calulation then yields the recurrence relation

$$\gamma_\tau^{(2n)}(0) = \sum_{r=0}^{n-1} \gamma_\tau^{(2n-2r-2)}(0) \sum_{k=0}^{r} \binom{2r}{2k} \gamma_\tau^{(2r-2k)}(0) v_\tau^{(2k)}(0) \tag{7}$$

for the even derivatives, whereas all odd derivatives vanish (see also Supplementary Eq. (4)). Up to third order in t, for example, we thus find

$$\begin{aligned} \gamma_\tau(t) &= 1 + \frac{v_\tau(0)}{2} t^2 + \mathcal{O}(t^4) \\ &= 1 - \frac{t^2}{2}\left[\left(\frac{F_1(\tau)}{\tau}\right)^2 v(0) - \left(\frac{F_2(\tau)}{\tau} - \frac{F_1(\tau)}{2}\right)^2 \ddot{v}(0)\right] \\ &\quad + \mathcal{O}(t^4)\,, \end{aligned} \tag{8}$$

illustrating how the initial decay of $\gamma_\tau(t)$ is controlled by the driving and the decay characteristics of $v(t)$.

Supplementary Note 2: Additional numerics

2.1. Fourier analysis and nonlinear response

As an interesting complementary, numerical characterization of the system's response to the periodic driving, we consider the Fourier transform

$$\tilde{a}(\omega) := \int_{-\infty}^{\infty} \mathrm{d}t\, a(t)\, \mathrm{e}^{-\mathrm{i}\omega t} \tag{9}$$

of the deviation of the time-evolved expectation values $\langle A\rangle_{\rho(t)}$ from the undriven thermal value A_{th},

$$a(t) := \langle A\rangle_{\rho(t)} - A_{\text{th}}\,. \tag{10}$$

More precisely speaking, we consider the discrete Fourier transformation of the numerically obtained time series $a(t)$ in an interval $[0, t_{\max}]$ with resolution (step size) Δt. For the example from Fig. 1e in the main paper, the corresponding Fourier spectra are shown in Supplementary Fig. 4.

Our first observation is that the periodic driving gives rise to delta peaks not only at the driving frequency but also at some of its higher harmonics, and that the remaining (smooth) part of the perturbed Fourier spectrum differs quite notably from its unperturbed counterpart. (A closer investigation of why the peaks at the second harmonics seem to be suppressed goes beyond the scope of our present work.) Both features indicate that the system's response to the periodic driving is outside the realm of what could be captured by a linear response theory.

Our second observation is that there are no indications of any additional delta peaks at noninteger multiples of the driving frequency, which might have been of interest with respect to the recent topic of time crystals, caused by a spontaneous breaking of the discrete time translation symmetry [18–24].

2.2. Temperature dependence

In the examples from the main paper (Figs. 1–3), the energy windows of the initial states were chosen sufficiently far away from the middle of the spectrum so that heating effects are not trivially absent, but the corresponding temperatures may still be perceived as relatively high. The reasons for these choices are mostly of technical nature to mitigate finite-size effects (see also Supplementary Note 2.3 below). As briefly mentioned in

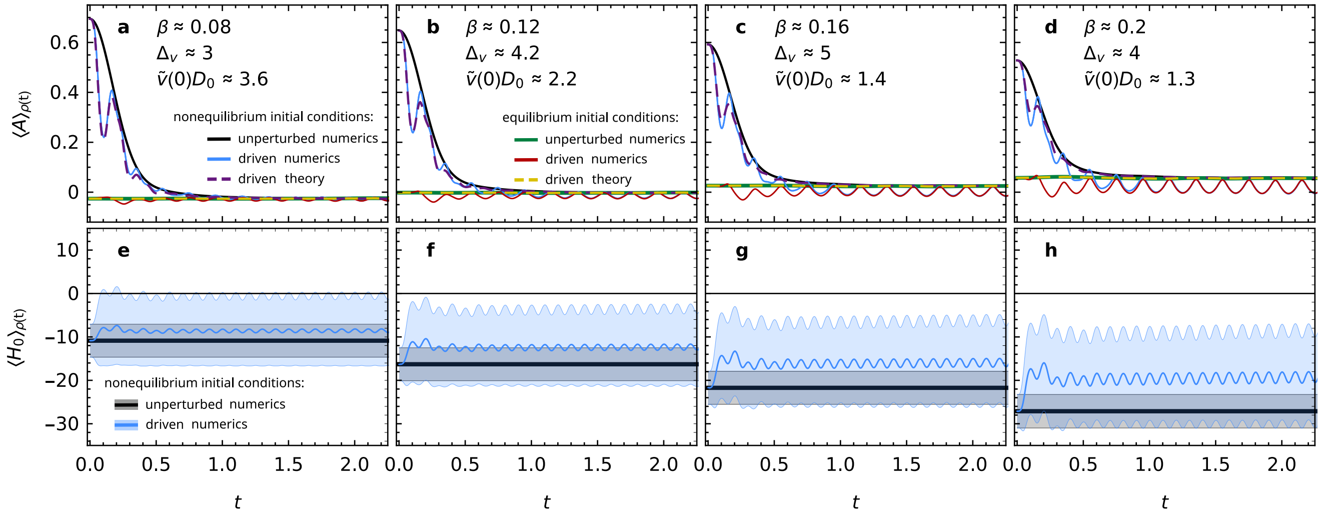

Supplementary Fig. 5. Time-dependent expectation values for the same two-dimensional spin lattice model as in Fig. 1 of the main paper with driving period $T = 0.2$ and amplitude $f_0 = 3.2$, using initial states of different inverse temperatures β as indicated in each column. Top (a-d): Dynamics of the magnetization correlation $A = \sigma^z_{2,2}\sigma^z_{3,3}$; bottom (e-h): Corresponding expectation values $\langle H_0\rangle_{\rho(t)}$ of the reference system's energy with bands indicating plus/minus one standard deviation $[\langle H_0^2\rangle_{\rho(t)} - \langle H_0\rangle^2_{\rho(t)}]^{1/2}$. In (e-h), only results for nonequilibrium initial conditions are shown; the curves for thermal equilibrium initial conditions are almost identical. Parameter values for $\beta \approx 0.08, 0.12, 0.16, 0.2$: $E = -12, -18, -24, -30$ (target energy of the Gaussian filter, see also Supplementary Fig. 6b); $A_{\rm th} = -0.026, -0.002, 0.026, 0.057$ (see also Supplementary Fig. 6a); $\tilde{v}(0)D_0 = 3.6, 2.25, 1.44, 1.3$ and $\Delta_v = 3.0., 4.2, 5.0, 4.0$ (see also third paragraph in "Interpretation and further examples" of the main paper).

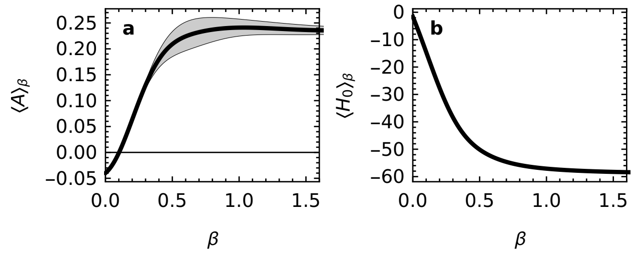

Supplementary Fig. 6. Thermal expectation values of $A = \sigma^z_{2,2}\sigma^z_{3,3}$ and H_0 in the two-dimensional spin lattice model from Fig. 1 of the main paper versus inverse temperature β. Depicted are estimates based on dynamical typicality methods [25–27], using imaginary-time propagation of three Haar-random states. Shaded region indicates one standard deviation of the mean (not visible in b).

the main paper (see "Theory" and "Methods" therein), the theory is based on the assumption that the initial energy window comprises a large number of energy levels with an approximately homogeneous density of states. These conditions are commonly satisfied best in the middle of the spectrum (highest density of states, smallest relative variations of the density of states), but the range of compatible temperatures is expected to increase with the system size due to the exponential growth of the overall number of levels as well as of the level density. Furthermore, we recall that the stalled-response effect is predicted to occur as long as energy absorption from the periodic driving is negligibile. The relative influence of this heating is typically smaller for higher temperatures, too, in the sense that the departure from the initially occupied energy window is smaller if all other parameters (notably T and f_0) are kept fixed (see also Supplementary Fig. 5). Given the limits of our computational resources, we therefore focused on relatively high temperatures in the main paper (see also the subsequent Supplementary Note 2.3).

To further substantiate these general arguments, we discuss the behavior for lower temperatures in Supplementary Fig. 5, using the same two-dimensional spin-lattice system as in Fig. 1 of the main paper. Likewise, the initial states are again generated according to Eq. (5) in the main paper, but now using successively lower target energies $E = -12$ (similar to Fig. 1), $-18, -24, -30$. As can be inferred from Supplementary Fig. 6b, these correspond to inverse temperatures $\beta \approx 0.08, 0.12, 0.16, 0.2$, respectively. This Supplementary Fig. 6b furthermore demonstrates that the energy in the ground state ($\beta \to \infty$) and at infinite temperature ($\beta = 0$) are indeed approximately -60 and -1, respectively, as stated in the main paper above Eq. (6).

Returning to Supplementary Fig. 5, the top panels (a)–(d) show the time-dependent expectation values of the magnetization correlation $A = \sigma^z_{2,2}\sigma^z_{3,3}$ as before. (Another example – starting from infinite temperature, $\beta = 0$ – can be found in Supplementary Fig. 9 below, cf. Supplementary Note 3.) To visualize the energy absorption in the driven system, we additionally show in the

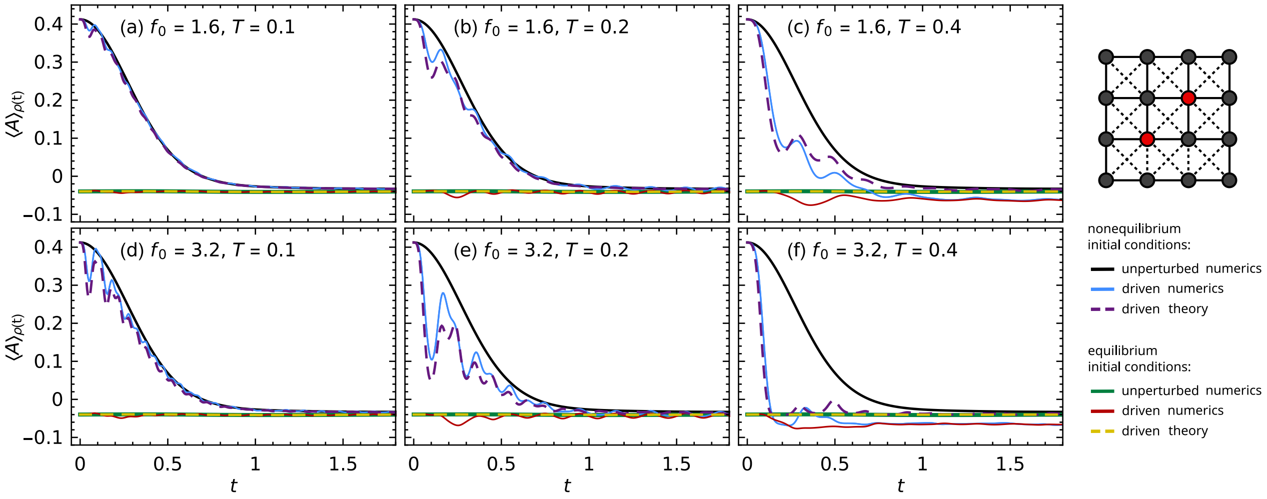

Supplementary Fig. 7. Same as in Fig. 1 of the main paper, but now for a 4×4 lattice (see also Supplementary Note 2.3 for more details).

bottom panels (e)–(h) the time-dependent expectation values $\langle H_0\rangle_{\rho(t)}$ of the unperturbed reference Hamiltonian H_0 (thick blue line) along with bands of one "standard deviation" $[\langle H_0^2\rangle_{\rho(t)} - \langle H_0\rangle_{\rho(t)}^2]^{1/2}$ (blue shaded region). For comparison, we also indicate the initially occupied energy window (black).

We observe that the driven system is not strictly confined to this initially occupied energy window in any of the examples from Supplementary Fig. 5. Instead, all of them show residual heating effects, which manifest themselves by a drift of the mean energy and broadening of the fluctuations, as expected for finite driving period. Notably, however, the departure from the initial energy window is smaller for states of higher temperature (smaller β). In line with this observation and the discussion in the main paper, the observable dynamics $\langle A\rangle_{\rho(t)}$ (top panels) shows stronger stalling effects for smaller β, too. We point out, however, that the response at very early times, namely the first minimum of $\langle A\rangle_{\rho(t)}$ at time $t \approx T/2 = 0.1$, is strongly suppressed for all displayed temperatures when comparing the thermal equilibrium initial conditions to the nonequilibrium behavior.

If all other parameters are kept fixed, stalled response is thus typically more pronounced at higher temperatures, and most pronounced at early times, because of relatively weaker heating effects, similarly as in the case of higher driving frequencies (cf. Supplementary Note 1.1). Stronger suppression at lower temperatures, in turn, can be expected upon increasing the driving frequency, or upon increasing the system sizes (see next subsection).

2.3. Finite-size effects

We recall that our square lattice model from Fig. 1 of the main paper only exhibits a relatively small extension of $L = 5$ sites along each of the two spatial directions. Hence, notable finite-size effects may still be expected.

To get an idea of their relevance, we consider a smaller version with $L = 4$ of the same two-dimensional spin-lattice system (cf. Eqs. (1), (3), and (4) in the main paper) as before. Again, we employ a sinusoidal driving protocol (Eq. (6) in the main paper) and initial states as in Eq. (5) of the main paper with $Q = \pi^+_{2,2}\pi^+_{3,3}$, but now choosing $E = -8$ and $\Delta E = 2$ as the target energy window in order to account for the different absolute energy scale of the $L = 4$ system, and to obtain the same inverse temperature $\beta \approx 0.08$ as in the example with $L = 5$. The thermal expectation value of our observable $A = \sigma^z_{2,2}\sigma^z_{3,3}$ in this window now assumes the value $A_{\rm th} = -0.040$.

As far as the theoretical prediction from Eqs. (9)–(10) of the main paper is concerned, an advantage of the smaller system size is that we can calculate the perturbation profile from Eq. (7) of the main paper directly by exact diagonalization. We find that it is well approximated by an exponential decay (see also Ref. [28]) of the form

$$\tilde{v}(E) = 5.08 \times 10^{-3}\, \mathrm{e}^{-|E|/8.4} \qquad (11)$$

for eigenstates of H_0 in the relevant energy window, $|E_\mu - E| \leq \Delta E$. Furthermore, the mean density of states in this window is $D_0 = 425$. The Fourier transform of $\tilde{v}(E)$, cf. Eq. (8) of the main paper, is thus

$$v(t) = \frac{36.3}{1 + (8.4\, t)^2}\,, \qquad (12)$$

from which $\gamma_\tau(t)$ can be calculated by integrating Eq. (10) of the main paper numerically as before.

The so-obtained numerical results along with the corresponding theoretical predictions are shown in Supplementary Fig. 7. As a technical aside, we note that in order to avoid additional finite-size artifacts from the employed dynamical-typicality method, we averaged over 100 random states $|\phi\rangle$ (cf. Eq. (5) of the main paper).

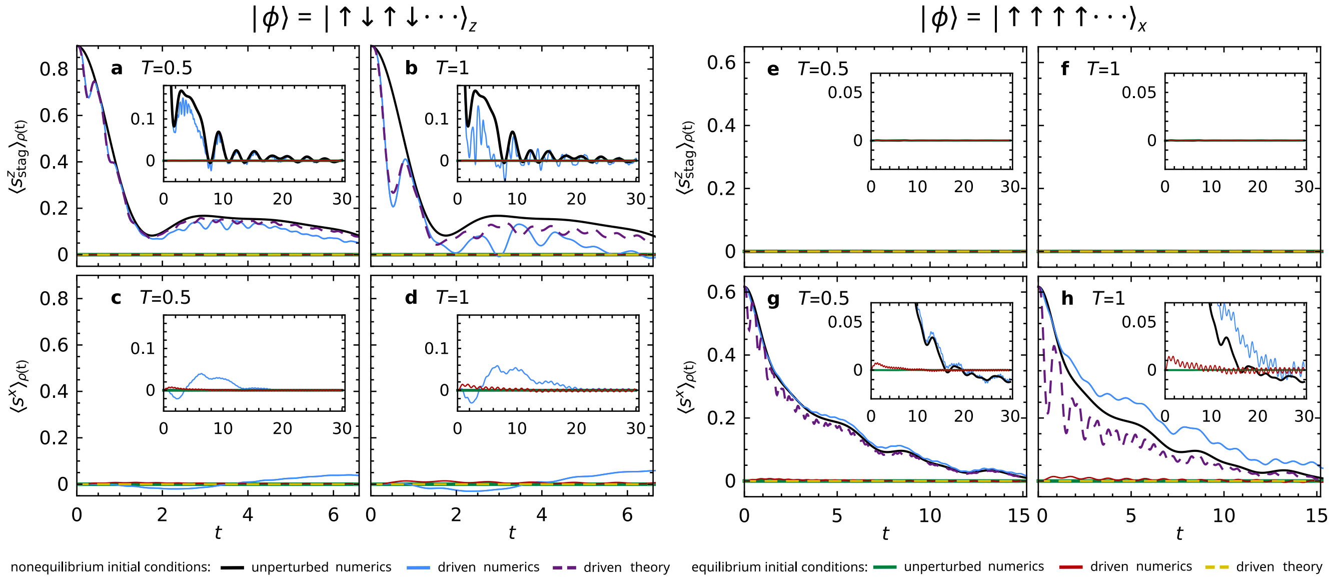

Supplementary Fig. 8. Time-dependent expectation values of s^z_{stag} from Supplementary Eq. (14) (top) and s^x from Supplementary Eq. (15) (bottom) for the nonintegrable transverse-field Ising model (cf. Fig. 2 of the main paper) with driving amplitude $f_0 = 4$, driving periods T as indicated in each panel, and two different initial states: Néel ordered in the z direction (left, a–d) and fully polarized in the x direction (right, e–h). All other parameters are as in Fig. 2 of the main paper. Insets: Same numerical data, but with rescaled x and y axes to display the long-time behavior. Often, some of the curves are hidden by others, most notably in panels (e) and (f).

Comparison of the these numerical results for $L = 4$ in Supplementary Fig. 7 with those for $L = 5$ in Fig. 1 of the main paper provides quite convincing evidence that the stalled response effect should become more pronounced as the system size is increased. In particular, the small remaining driving effects in case of thermal equilibrium initial conditions (red curves) may be expected to become still smaller upon further increasing L, which, however, is computationally infeasible for us in practice. Furthermore, we observe that the theoretical prediction according to Eq. (9) from the main paper agrees better with the numerics (solid lines) for $L = 5$ than for $L = 4$, in accordance with the fact that the derivation of the theory assumes large system sizes (see also "Methods" in the main paper).

Finally, we also note that increasing β (cf. Supplementary Note 2.2) seems to have somewhat similar qualitative effects as decreasing the system size L. For any given such β, this suggests once again that the agreement between numerics and theory, and thus the manifestation of stalled response, would significantly improve if one increased L further.

2.4. *Special case $A = V$*

As explained in the "Methods" of the main paper, the typicality framework adopted to derive our main analytical result, Eq. (9) in the main paper, is not well suited to describe situations in which the observable A is strongly correlated with the driving operator V. To illustrate this and to clarify whether the effect of stalled response per se still applies (as supported by the heuristic arguments provided in the main paper's "Basic physical mechanisms" section), we investigate the case $A = V$ in the following. Since V is an extensive operator, it is appropriate to consider initial states that are globally out of equilibrium (otherwise the observable $A = V$ is expected to exhibit no difference compared to equilibrium initial states for asymptotically large systems). Let us therefore return to our example of the nonintegrable Ising model from Fig. 2 of the main paper, prepared in a (Gaussian-filtered) Néel state,

$$|\psi\rangle \propto \mathrm{e}^{-(H_0-E)^2/4\Delta E^2}\,|\phi\rangle \tag{13}$$

with $|\phi\rangle = |{\uparrow\downarrow\uparrow\downarrow}\cdots\rangle_z$. The subscript '$z$' here explicitly indicates that the state is expressed with respect to the spin basis in the z direction. We also compare the resulting dynamics to the one obtained from the corresponding thermal equilibrium conditions, emulated as before by choosing $|\phi\rangle$ to be a Haar-random state in Supplementary Eq. (13). In both cases, we furthermore employ in Supplementary Eq. (13) the same parameters $E = -2.4$ and $\Delta E = 1$ as in Fig. 2 of the main paper.

A natural observable in view of the initial state's Néel order is the staggered magnetization in the z direction,

$$s^z_{\text{stag}} := \frac{1}{L}\sum_{j=1}^{L}(-1)^{j+1}\sigma^z_j\,. \tag{14}$$

As anticipated from its close connection to the single-site magnetization σ^z_1 shown in Fig. 2a of the main pa-

per, the numerics for $s^z_{\rm stag}$ in Supplementary Fig. 8a–b again exhibits stalled response and good agreement with our analytical prediction, Eq. (9) of the main paper, for sufficiently small times.

Next we turn to Supplementary Fig. 8c–d showing the time evolution of the x magnetization,

$$s^x := \frac{1}{L} \sum_{j=1}^{L} \sigma^x_j \, . \tag{15}$$

Up to a trivial factor $-1/L$, which we henceforth ignore, this observable $A = s^x$ thus coincides with the driving operator V in our present setup (see also the caption of Fig. 2 in the main paper).

Our first observation is that, numerically, the initial response of s^x away from equilibrium is weaker than what is seen in $s^z_{\rm stag}$. (Note that we deliberately chose the same y-axis scaling for the plots in panels (a) through (d) to facilitate their direct comparison.)

Second, the numerically observed reaction to the driving is significantly stronger away from equilibrium than near thermal equilibrium, as can be seen by comparing the solid blue and red lines in the insets in particular. In other words, the stalled-response phenomenon also manifest itself for the special observable $A = V$.

Third, the latter applies despite the fact that the theoretical prediction from Eq. (9) of the main paper breaks down for $A = V$, as anticipated there and above. Indeed, since the thermal expectation value $s^x_{\rm th} = 0$ and the unperturbed dynamics $\langle s^x \rangle_{\rho_0(t)} = 0$ (the associated black lines in Supplementary Fig. 8c–d are hidden behind the green ones), the theory from Eq. (9) of the main paper predicts $\langle s^x \rangle_{\rho(t)} = 0$ for the driven dynamics, too (the dashed purple lines are likewise hidden behind the green solid ones), while the actually observed numerical response quite notably deviates from this prediction. The same theoretical predictions also apply to the thermal equilibrium initial conditions, but here the numerically observed deviations from the theory are less severe due to the occurrence of stalling.

Since the unperturbed dynamics for $A = V$ with Néel-ordered initial conditions are trivial, we consider a second example where the initial state is of the same form (cf. Supplementary Eq. (13)), but with $|\phi\rangle = |{\uparrow\uparrow}\cdots\rangle_x$ being fully polarized in the x direction. Hence, we obtain an initial value $\langle s^x \rangle_{\rho(0)}$ that is far from the thermal equilibrium value $s^x_{\rm th} = 0$ (see Supplementary Fig. 8g–h).

On the other hand, the unperturbed dynamics of the staggered magnetization is now trivial, $\langle s^z_{\rm stag} \rangle_{\rho_0(t)} = (s^z_{\rm stag})_{\rm th} = 0$, for both nonequilibrium and equilibrium initial conditions (cf. Supplementary Fig. 8e–f; black lines hidden behind green ones). Hence the theory from Eq. (9) of the main paper predicts $\langle s^z_{\rm stag} \rangle_{\rho(t)} = 0$ in both cases. This time, this theoretically predicted behavior is indeed found in the actual dynamics of $s^z_{\rm stag}$, i.e., one essentially observes no response in Supplementary Fig. 8e–f for both the nonequilibrium initial state (blue lines hidden behind green ones) and the equilibrium one (red lines).

For $A = V$ in Supplementary Fig. 8g–h, by contrast, the theory fails to predict the correct dynamics in the nonequilibrium setting (solid blue vs. dashed purple lines), but as emphasized repeatedly, this is understood to result from the adopted typicality framework in the derivation of Eq. (9) of the main paper. Notwithstanding, we observe that the difference between the unperturbed and driven dynamics is overall larger when the system is away from equilibrium (black and blue lines) compared to the situation close to thermal equilibrium (green and red lines). Therefore, despite the breakdown of our analytical theory, the stalled-response effect itself remains observable, as supported additionally by the heuristic arguments provided in the main paper's "Basic physical mechanisms" section.

Supplementary Note 3: Relating $v(t)$ to two-point correlation functions

Here, we discuss the connection between the two-point correlation function $\langle V(t) V \rangle_{\rho_{\rm mc}}$ and the function $v(t)$, which affects the observable response via Eq. (10) of the main paper. According to its definition in Eq. (8) of the main paper, $v(t)$ is the Fourier transform of the perturbation profile $\tilde{v}(E) = [|V_{\mu\nu}|^2]_E$ from Eq. (7) there. The local energy average $[|V_{\mu\nu}|^2]_E$ as introduced in and around this Eq. (7) can be expressed more formally as

$$[|V_{\mu\nu}|^2]_E = \sum_{\mu,\nu \in S_\Delta} k_E(E_\mu, E_\nu) \, |V_{\mu\nu}|^2 \, , \tag{16}$$

where $k_E(E_\mu, E_\nu)$ is a suitable averaging kernel which enforces the condition $|E_\mu - E_\nu| \approx E$ and satisfies $\sum_{\mu,\nu \in S_\Delta} k_E(E_\mu, E_\nu) = 1$. Moreover, S_Δ is the set of all indices such that $E_\mu \in \Delta$, i.e., all states whose energy lie in the initially occupied microcanonical energy window Δ.

Substituting Supplementary Eq. (16) into Eq. (8) from the main paper, we obtain

$$v(t) = \sum_{\mu,\nu \in S_\Delta} |V_{\mu\nu}|^2 \int \mathrm{d}E \, D_0 \, k_E(E_\mu, E_\nu) \, \mathrm{e}^{\mathrm{i}Et} \, . \tag{17}$$

For concreteness, let us now choose $k_E(E_\mu, E_\nu) \simeq \delta(|E_\mu - E_\nu| - E) \, / \, 2|S_\Delta| D_0$, where $|S_\Delta| = \int_\Delta \mathrm{d}E \, D_0$ is the number of levels in Δ. Adopting this form in Supplementary Eq. (17), we obtain

$$v(t) \simeq \frac{1}{2|S_\Delta|} \sum_{\mu,\nu \in S_\Delta} |V_{\mu\nu}|^2 \mathrm{e}^{\mathrm{i}(E_\mu - E_\nu)t} = \frac{1}{2|S_\Delta|} \, \mathrm{tr}[P V(t) P V] \, , \tag{18}$$

where $V(t) := \mathrm{e}^{\mathrm{i}H_0 t} V \mathrm{e}^{-\mathrm{i}H_0 t}$ and $P := \sum_{\mu \in S_\Delta} |\mu\rangle\langle\mu|$ is the projector onto Δ. Observing that the microcanonical ensemble is given by $\rho_{\rm mc} = P/|S_\Delta|$, we can finally rewrite $v(t)$ as

$$v(t) \simeq \frac{1}{2} \langle V(t) P V \rangle_{\rho_{\rm mc}} \, . \tag{19}$$

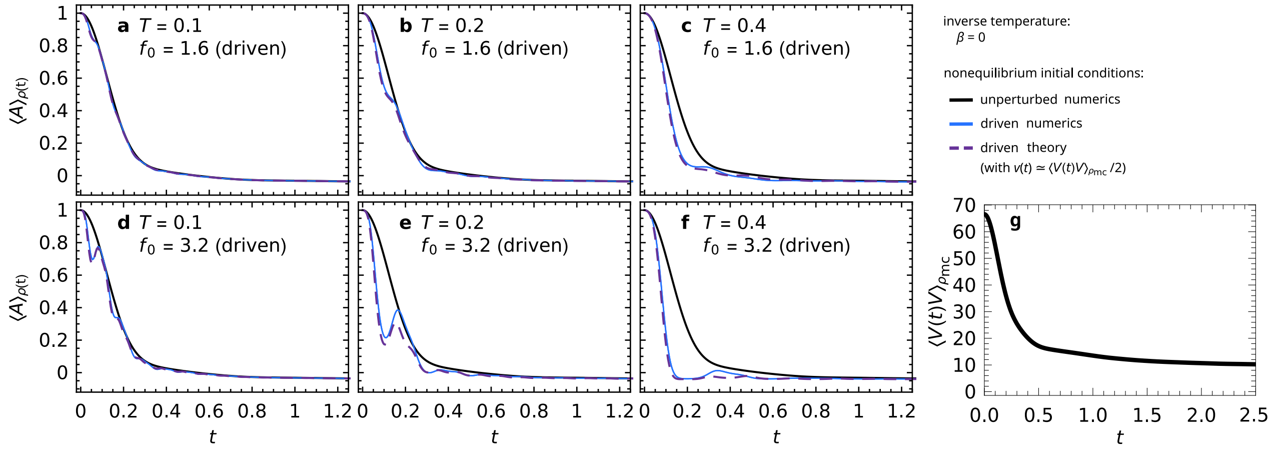

Supplementary Fig. 9. (a-f) Time-dependent expectation values $\langle A\rangle_{\rho(t)}$ of the magnetization correlation $A = \sigma^z_{2,2}\sigma^z_{3,3}$ for the 5×5 lattice spin system from Eqs. (1), (3), (4), (6) of the main paper (see also Fig. 1 there) at infinite temperature ($\beta = 0$). Solid lines: numerical results for nonequilibrium initial conditions from Supplementary Eq. (21) for driving amplitudes $f_0 = 0$ (unperturbed, black), and for driving periods T and amplitudes f_0 as indicated in each panel (driven, blue). Dashed lines: corresponding theoretical prediction from Eq. (9) of the main paper, where $|\gamma_t(t)|^2$ is obtained as the solution of Eq. (10) of the main paper using the approximation from Supplementary Eq. (20) with the numerically obtained two-point correlation function $\langle V(t)V\rangle_{\rho_{\rm mc}}$ from Supplementary Eq. (22) as shown in (g).

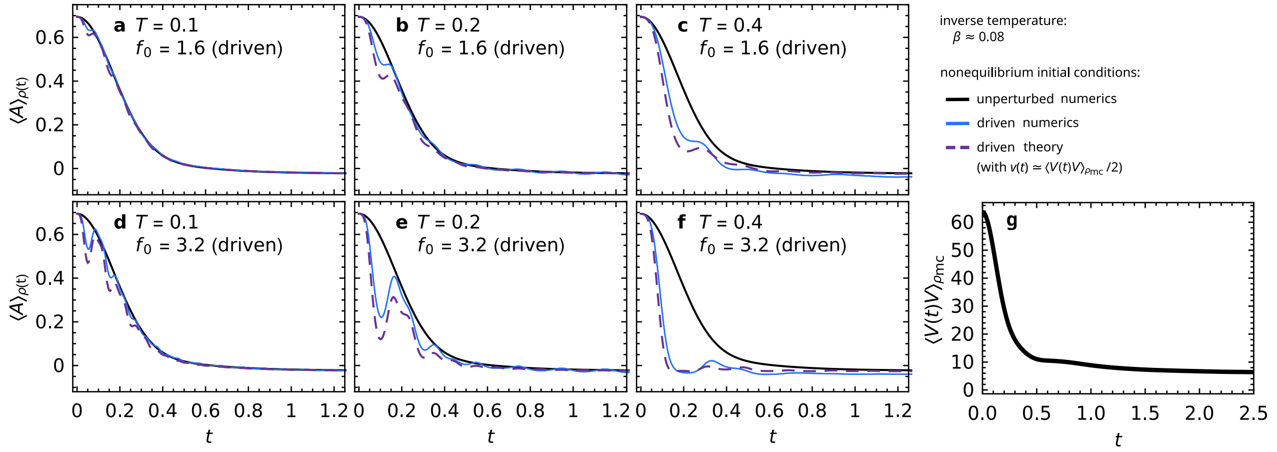

Supplementary Fig. 10. Same as in Supplementary Fig. 9, but now for $\beta \approx 0.08$; see also Supplementary Note 3 for more details.

The right-hand side is reminiscent of the two-point correlation function $\langle V(t)V\rangle_{\rho_{\rm mc}}/2$, but in general not identical to it because of the additional projector P between the factors of V, which effectively restricts the domain of the matrix product. However, the projector P approaches the identity operator as the temperature is increased.

Altogether, these non-rigorous arguments thus suggest that it may be possible to employ the approximation

$$v(t) \simeq \langle V(t)V\rangle_{\rho_{\rm mc}}/2 \tag{20}$$

at sufficiently high temperatures.

To test this conjecture, we return to the example setup from Fig. 1 of the main paper. We remove the Gaussian filter in the initial conditions (cf. Eq. (5) of the main paper), such that the resulting initial state is effectively at infinite temperature,

$$|\psi\rangle \propto \pi^+_{2,2}\pi^+_{3,3}|\phi\rangle\,, \tag{21}$$

where $|\phi\rangle$ is a Haar-random state in the $S^z = -1$ magnetization subsector as before. The solid lines in Supplementary Fig. 9a-f show the corresponding numerically obtained dynamics for the unperturbed (black) and driven (blue) systems.

Furthermore, we numerically calculate the two-point correlation function $\langle V(t)V\rangle_{\rho_{\rm mc}}$ at infinite temperature ($\rho_{\rm mc} = \mathbb{1}/\operatorname{tr}(\mathbb{1})$) using dynamical typicality [25–27]. Concretely, we approximate

$$\langle V(t)V\rangle_{\rho_{\rm mc}} \simeq \langle\phi_{\mathbb{1}}(t)|V|\phi_V(t)\rangle\,, \tag{22}$$

where $|\phi_Q(t)\rangle := \mathrm{e}^{-\mathrm{i}H_0 t}|\phi_Q\rangle$, $|\phi_Q\rangle := Q|\phi\rangle$, and $|\phi\rangle$ is a

Haar-random state as before. The so-obtained correlation function is shown in Supplementary Fig. 9g.

Next we use this result to substitute $v(t) = \langle V(t)V\rangle_{\rho_{\rm mc}}/2$ in the integro-differential equation (10) from the main paper for $\gamma_\tau(t)$. Integrating the equation numerically as before and utilizing the solution for $|\gamma_t(t)|^2$ in the prediction from Eq. (9) of the main paper, we finally obtain the dashed purple lines in Supplementary Fig. 9a-f. The agreement between theory and numerics is remarkably good throughout the inspected range of amplitudes and driving periods.

As an illustration of finite-temperature effects in (20), we repeat the entire procedure for the identical setup as in Fig. 1 from the main paper, meaning that the initial state is now again of the same form as in Eq. (5) of the main paper with target energy $E = -12$ and width $\Delta E = 4$ (such that $\beta \approx 0.08$). The corresponding numerical results (solid lines) in Supplementary Fig. 10a-f are thus identical to those in Fig. 1 of the main paper. To estimate the time-correlation function $\langle V(t)V\rangle_{\rho_{\rm mc}}$, we follow the dynamical-typicality approach from Supplementary Eq. (22) again, but choose $|\phi_Q\rangle := Q\mathrm{e}^{-(H_0-E)/4\Delta E^2}|\phi\rangle$ now, thereby emulating the microcanonical density operator of the energy window around the target energy $E = -12$. This yields Supplementary Fig. 10g.

The dashed lines in Supplementary Fig. 10a-f are then again obtained by employing the approximation from Supplementary Eq. (20) in Eq. (10) of the main paper. Comparing theory and numerics, we observe stronger deviations than in the infinite-temperature case, but we still recover the qualitative features of the response and even achieve reasonable quantitative proximity.

Supplementary Note 4: Relation to Floquet theory

Numerous insights about the long-time behavior of periodically driven systems, including heating effects, metastable plateau regimes ("Floquet prethermalization"), and their topological properties, have been obtained using so-called Floquet theory; see, for example, Refs. [1–8, 10–13, 15, 17, 24, 29–38] and references therein. As explained in the main paper, the focus of our present study is on the complementary regime of short-to-intermediate times. Crucially, our methodological approach is distinct from traditional Floquet theory, too. The following discussion is to clarify their relationship.

Floquet theory is based on the mathematical insight that solutions for the propagator $\mathcal{U}(t)$ of the time-dependent Schrödinger equation $\frac{\mathrm{d}}{\mathrm{d}t}\mathcal{U}(t) = -\mathrm{i}H(t)\mathcal{U}(t)$ can be decomposed as [33, 39, 40]

$$\mathcal{U}(t) = \mathcal{M}(t)\,\mathrm{e}^{-\mathrm{i}H_{\rm F}t} \tag{23}$$

if $H(t) = H(t+T)$ is time periodic. Here $H_{\rm F}$ is a time-independent Hermitian operator, the so-called Floquet Hamiltonian. Furthermore, $\mathcal{M}(t)$ is a time-dependent unitary operator of the same periodicity as the driving protocol, $\mathcal{M}(t+T) = \mathcal{M}(t)$, sometimes called the micromotion operator.

Since $\mathcal{U}(0) = \mathbb{1}$, one immediately concludes $\mathcal{M}(nT) = \mathbb{1}$ for all $n \in \mathbb{Z}$ and thus $\mathcal{U}(nT) = \mathrm{e}^{-\mathrm{i}H_{\rm F}nT}$. In other words, the dynamics generated by the time-independent Floquet Hamiltonian $H_{\rm F}$ agrees with the dynamics of the actual system (generated by the time-dependent Hamiltonian $H(t)$) at integer multiples of the driving period T. (As an aside, the reference time can be chosen arbitrarily, i.e., by a suitable adaptation of $H_{\rm F}$, one can instead achieve agreement for all $t_n = nT + \theta$ with an arbitrary, but fixed $\theta \in [0, T)$. Among other things, this implies that the choice of $H_{\rm F}$ is not unique, but these technical details are not important for the ensuing discussion.)

Exploiting the stroboscopic agreement between the dynamics generated by $H_{\rm F}$ and $H(t)$, the vast majority of studies investigating the long-time behavior focused on properties of the Floquet Hamiltonian $H_{\rm F}$ and largely ignored the periodic modulations induced by the micromotion operator $\mathcal{M}(t)$. For example, "Floquet prethermalization" [6, 11–13, 15, 17, 35, 37] describes the observation that, for sufficiently small driving periods T, the dynamics generated by $H_{\rm F}$ resembles ordinary "prethermalization" [41, 42]: The system relaxes from its initial nonequilibrium state to some quasistationary intermediate state, and the true thermal equilibrium state is only approached at much later times. In the Floquet case, the quasistationary intermediate state arises as a result of the strong suppression of heating at fast driving. Crucially, the system can thus spend long times close to this nontrivial intermediate state, before heating eventually takes it towards the featureless infinite-temperature ("thermal equilibrium") state.

The periodic modulations by the micromotion operator $\mathcal{M}(t)$ are usually disregarded when discussing this effect. Nevertheless, $\mathcal{M}(t)$ can generally still induce a strong time dependence of observable expectation values, even if the stroboscopic dynamics generated by $H_{\rm F}$ relaxes to a plateau value. An example is provided in Fig. 3 of the main paper, where the stroboscopic dynamics becomes stationary, but $\langle A\rangle_{\rho(t)}$ continues to oscillate.

Our principal observation, the phenomenon of stalled response, implies that those periodic modulations are also suppressed if the accompanying unperturbed system finds itself near thermal equilibrium and heating is negligible. Put differently, the micromotion operator $\mathcal{M}(t)$ affects states far away from equilibrium more strongly than states close to thermal equilibrium. Coming back to the example from Fig. 3 of the main paper once again, we recall that the unperturbed system there relaxes to an equilibrium state. Crucially, however, this state is different from the thermal equilibrium state of the full system, and therefore $\mathcal{M}(t)$ continues to have an effect even though the $H_{\rm F}$ dynamics has settled down.

From a technical point of view, the reason why we can characterize the response continuously in time rather

than stroboscopically is that we do *not* adopt a decomposition like in Supplementary Eq. (23). Instead, we always work with the full propagator $\mathcal{U}(t)$, particularly when employing the Magnus expansion according to Eq. (14) in the main paper.

On the other hand, the fact that we truncate the Magnus expansion at second order means that our characterization of the "prethermal" plateau state is less accurate than state-of-the-art results obtained from high-frequency expansions of stroboscopic Hamiltonians (such as the Floquet Hamiltonian) [11–13, 15, 35]. Within our approximation, and if we tacitly assume that $f(t)$ averages to zero over one driving period, the plateau is essentially determined by the time-averaged Hamiltonian H_0, see also the "Limits of applicability" subsection in the Methods. This is primarily relevant for the long-time expectation value A_{th} in the prediction from Eq. (9) of the main paper. In light of the literature on stroboscopic dynamics and observing that H_0 is the first-order approximation of H_{F}, higher-order corrections could shift this value. On the other hand, it was argued in Ref. [12] that these corrections will generically be small and that the prethermal plateau state is well approximated by the microcanonical ensemble of H_0. In any case, assuming such a shift in Eq. (9) from the main paper would give room for small remnant oscillations between the plateau value prescribed by the Floquet Hamiltonian and the thermal value approached by the unperturbed dynamics $\langle A\rangle_{\rho_0(t)}$. We emphasize, however, that higher-order corrections will also change the "response function" $\gamma_\tau(t)$ and may even affect the overall structure of the prediction.

Supplementary Note 5: Driving-operator ensembles

The typicality approach employed in this work (cf. Methods in the main paper) covers statistical ensembles of driving operators V of the following general form: The distributions are expressed in terms of probability densities for the matrix representation $V_{\mu\nu} := {}_0\langle\mu|V|\nu\rangle_0$ in the eigenbasis of the reference Hamiltonian H_0. These matrix elements are assumed to be statistically independent apart from Hermiticity ($V_{\mu\nu} = V^*_{\nu\mu}$). The probability density $p(V)$ of V with respect to the Lebesgue measure $[\mathrm{d}V] := [\prod_\mu \mathrm{d}V_{\mu\mu}][\prod_{\mu<\nu} 2\mathrm{d}(\mathrm{Re}\, V_{\mu\nu})\mathrm{d}(\mathrm{Im}\, V_{\mu\nu})]$ can therefore be written in the form

$$p(V) = \prod\nolimits_{\mu\leq\nu} p_{\mu\nu}(V_{\mu\nu}) \tag{24}$$

with $p_{\mu\nu}(v) := \mathbb{E}[\delta(v - V_{\mu\nu})]$. (We recall that $\mathbb{E}[\cdots]$ denotes the average over the V ensemble.) The marginal probability densities are of the form $p_{\mu\nu}(v) = p_{|E_\mu - E_\nu|}(v)$, where $\{p_E(v)\}_{E>0}$ is a family of probability densities on $\mathbb{C}$ with mean zero and variance $\tilde{v}(E)$ (cf. Eq. (7) in the main paper), and $p_0(v)$ is a probability density on $\mathbb{R}$ with mean zero and variance $\tilde{v}(0)$. We furthermore assume that the statistical properties of the $V_{\mu\nu}$ are unbiased with respect to the choice of the (unphysical) phase of the eigenvectors $|\mu\rangle_0$, meaning that $p_E(v)$ only depends on the absolute value $|v|$.

Note that this automatically implies the vanishing mean for $E > 0$ ($\mu \neq \nu$). The assumption of a vanishing mean of the distribution $p_0(v)$ (i.e., of the diagonal matrix elements), in turn, constitutes no loss of generality because any bias could be gauged away by adding a constant (proportional to the identity) to H_0, which does not alter the dynamics. In light of the generalized central limit theorem effective below (see Supplementary Note 6 and the discussion below Supplementary Eq. (36) in particular), the considered classes include essentially all reasonable, unbiased distributions $p_E(v)$ for the matrix elements $V_{\mu\nu}$ which are compatible with the perturbation profile $\tilde{v}(E)$ from Eq. (7) of the main paper.

In terms of the distribution $p(V)$ from Supplementary Eq. (24), the ensemble average $\mathbb{E}[\cdots]$ can thus be written explicitly as

$$\mathbb{E}[\cdots] \equiv \int [\mathrm{d}V]\, \cdots\, p(V)\,. \tag{25}$$

Supplementary Note 6: Ensemble-averaged auxiliary dynamics

In the following, we evaluate the ensemble-averaged auxiliary dynamics $\mathbb{E}[\langle A\rangle_{\rho(t,\tau)}]$, i.e., we establish Eqs. (22)–(24) from the main paper. We focus on an arbitrary but fixed $\tau > 0$. The time-dependent expectation values of the observable A in the state $\rho(t,\tau)$, obtained by evolving $\rho(0)$ for the time t with the auxiliary Hamiltonian $H^{(\tau)}$ from Eqs. (19) and (20) of the main paper, can then be written as

$$\langle A\rangle_{\rho(t,\tau)} = \sum_{\substack{\mu_1,\mu_2,\\ \nu_1,\nu_2}} \rho_{\mu_1\nu_2}(0) A_{\mu_2\nu_1} \times \sum_{m,n} \mathrm{e}^{\mathrm{i}(E_n^{(\tau)} - E_m^{(\tau)})t} U^{(\tau)}_{m\mu_1} U^{(\tau)}_{n\mu_2} U^{(\tau)*}_{m\nu_1} U^{(\tau)*}_{n\nu_2}\,. \tag{26}$$

Here $E_n^{(\tau)}$ denotes the eigenvalue of $H^{(\tau)}$ corresponding to the eigenvector $|n(\tau)\rangle$ and $U^{(\tau)}_{n\mu} := \langle n(\tau)|\mu\rangle_0$ are the overlaps between those $|n(\tau)\rangle$ and the eigenvectors $|\mu\rangle_0$ of the unperturbed reference Hamiltonian H_0. Hence, the evaluation of $\mathbb{E}[\langle A\rangle_{\rho(t,\tau)}]$ requires calculating ensemble averages over four factors of eigenvector overlaps $U^{(\tau)}_{n\mu}$. In addition, averages over eight such factors will be needed later in Supplementary Note 8 to determine the average $\mathbb{E}[\xi(t,\tau)^2]$ of the fluctuations $\xi(t,\tau)$, cf. above Eq. (25) in the main paper. By a suitable extension of the methods developed in the Supplemental Material of Ref. [43], these fourth and eighth moments can be traced back to combinations of the second moment

$$\mathbb{E}[|U^{(\tau)}_{n\mu}|^2] =: u(E_n - E_\mu, \tau) \tag{27}$$

with the function $u(E,\tau)$ still to be determined. Adopting those results, one finds that the ensemble-averaged auxiliary dynamics satisfies

$$\mathbb{E}[\langle A\rangle_{\rho(t,\tau)}] = \langle A\rangle_{\tilde\rho(\tau)} + |\gamma_\tau(t)|^2 \left[\langle A\rangle_{\rho_0(t)} - \langle A\rangle_{\tilde\rho(\tau)}\right] , \quad (28)$$

where

$$\gamma_\tau(t) := \int \mathrm{d}E\, D_0\, \mathrm{e}^{\mathrm{i}Et}\, u(E,\tau) , \quad (29)$$

$$\tilde\rho(\tau) := \sum_{\mu,\nu} \rho_{\mu\mu}(0)\, \tilde u(E_\mu - E_\nu, \tau) |\nu\rangle_{00}\langle\nu| , \quad (30)$$

$$\tilde u(E,\tau) := \int \mathrm{d}E'\, D_0\, u(E-E',\tau)\, u(E',\tau) . \quad (31)$$

Here D_0 is the density of states of the reference Hamiltonian H_0 in the energy window Δ as introduced above Eq. (7) of the main paper.

The remaining step thus consists of evaluating and interpreting the second moment $\mathbb{E}[|U^{(\tau)}_{n\mu}|^2] = u(E_n - E_\mu, \tau)$ from Supplementary Eq. (27). Introducing the resolvent or Green's function

$$\mathcal{G}^{(\tau)}(z) := (z - H^{(\tau)})^{-1} \quad (32)$$

of $H^{(\tau)}$, this second moment can be written as [44, 45]

$$\mathbb{E}[|U^{(\tau)}_{n\mu}|^2] = \frac{\lim_{\eta\to 0+} \mathbb{E}\left[\mathcal{G}^{(\tau)}_{\mu\mu}(E^{(\tau)}_n - \mathrm{i}\eta) - \mathcal{G}^{(\tau)}_{\mu\mu}(E^{(\tau)}_n + \mathrm{i}\eta)\right]}{2\pi\mathrm{i}D_0} \quad (33)$$

with $\mathcal{G}^{(\tau)}_{\mu\nu}(z) := {}_0\langle\mu|\mathcal{G}^{(\tau)}(z)|\nu\rangle_0$. We focus on a Hilbert space of finite dimension $N \gg 1$ (e.g., the energy window from above Eq. (7) in the main paper). Employing so-called supersymmetry methods [44–48], those matrix elements $\mathcal{G}^{(\tau)}_{\mu\nu}(z)$ of the resolvent can be expressed as a Gaussian integral over commuting (complex-valued) variables x_α and anticommuting (Grassmann) variables χ_α ($\alpha = 1, \ldots, N$), which we collect in a supervector $X := (X_1^\mathsf{T} \cdots X_N^\mathsf{T})^\mathsf{T}$ with $X_\alpha := (x_\alpha\ \chi_\alpha)^\mathsf{T}$. Denoting the associated integration measure by $[\mathrm{d}X\mathrm{d}X^*] := \prod_\alpha \mathrm{d}x_\alpha \mathrm{d}x^*_\alpha \mathrm{d}\chi_\alpha \mathrm{d}\chi^*_\alpha$ and defining the diagonal matrices $L^\pm := \mathrm{diag}(\pm 1, 1)$ as well as the shorthand $z^\pm := E \pm \mathrm{i}\eta$ with $E \in \mathbb{R}$, $\eta > 0$, we can then write

$$\mathcal{G}^{(\tau)}_{\mu\nu}(z^\pm) = \mp\mathrm{i} \int \frac{[\mathrm{d}X\mathrm{d}X^*]}{(\mp 2\pi)^N} x_\mu x^*_\nu\, \mathrm{e}^{\mathrm{i}X^\dagger[(z^\pm - H^{(\tau)})\otimes L^\pm]X} . \quad (34)$$

To average over the perturbation ensemble, we inspect the part of the exponent in Supplementary Eq. (34) that depends on the random variables $V_{\mu\nu}$. Employing the definition from Eq. (20) of the main paper, we obtain

$$\mathrm{e}^{-\mathrm{i}X^\dagger(V^{(\tau)}\otimes L^\pm)X} = \mathrm{e}^{-\mathrm{i}\sum_\alpha \lambda^{(\tau)}_{\alpha\alpha} X^\dagger_\alpha L^\pm X_\alpha V_{\alpha\alpha} - \mathrm{i}\sum_{\alpha<\beta}(\mathrm{Re}\,V_{\alpha\beta})(\lambda^{(\tau)}_{\alpha\beta} X^\dagger_\alpha L^\pm X_\beta + \text{c.c.}) - \mathrm{i}\sum_{\alpha<\beta}\mathrm{i}(\mathrm{Im}\,V_{\alpha\beta})(\lambda^{(\tau)}_{\alpha\beta} X^\dagger_\alpha L^\pm X_\beta - \text{c.c.})} \quad (35)$$

where "c.c." indicates the complex conjugate of the preceding term and

$$\lambda^{(\tau)}_{\alpha\beta} := \frac{F_1(\tau)}{\tau} - \mathrm{i}(E_\alpha - E_\beta)\left[\frac{F_2(\tau)}{\tau} - \frac{F_1(\tau)}{2}\right] . \quad (36)$$

Recalling the definition of the perturbation ensembles from Supplementary Note 5, the exponent in Supplementary Eq. (35) is thus a sum of N^2 independent and unbiased random variables. Adopting the central limit theorem, this sum approaches an unbiased normal distribution as $N \to \infty$, whose variance is given by the sum of variances of the individual terms, regardless of further details of the distributions $p_E(v)$ from below Supplementary Eq. (24). For sufficiently large N, we can therefore take all $p_E(v)$ to be, for example, Gaussian distributions with mean zero and variance $\tilde v(E)$ because they lead to the same limiting distribution in Supplementary Eq. (35), i.e., we can adopt, without loss of generality,

$$p_0(v) = \frac{\mathrm{e}^{-v^2/2\tilde v(0)}}{\sqrt{2\pi\tilde v(0)}} \quad \text{and} \quad p_{E>0}(v) = \frac{\mathrm{e}^{-|v|^2/\tilde v(E)}}{\pi\tilde v(E)} . \quad (37)$$

Evaluating the ensemble average of Supplementary Eq. (35) according to Supplementary Eq. (25) then amounts to computing a product of N^2 one-dimensional Gaussian integrals. Substituting the result into Supplementary Eq. (34) yields

$$\mathbb{E}[\mathcal{G}^{(\tau)}_{\mu\nu}(z^\pm)] = \mp\mathrm{i}\int \frac{[\mathrm{d}X\mathrm{d}X^*]}{(\mp 2\pi)^N} x_\mu x^*_\nu\, \mathrm{e}^{-\frac{1}{2}\sum_{\alpha,\beta}|\lambda^{(\tau)}_{\alpha\beta}|^2 \tilde v(E_\alpha - E_\beta)\,\mathrm{str}(X_\alpha X^\dagger_\alpha L^\pm X_\beta X^\dagger_\beta L^\pm) + \mathrm{i}\sum_\alpha (z^\pm - E_\alpha) X^\dagger_\alpha L^\pm X_\alpha} , \quad (38)$$

where $\mathrm{str}(\cdots)$ denotes the supertrace. Next we perform a supersymmetric Hubbard-Stratonovich transformation [45, 47] to rewrite the exponential of the fourth-order term in X as a superintegral involving only quadratic terms in X, namely

$$\mathrm{e}^{-\frac{1}{2}\sum_{\alpha,\beta} s_{\alpha\beta}\,\mathrm{str}(X_\alpha X^\dagger_\alpha L^\pm X_\beta X^\dagger_\beta L^\pm)} = \int \frac{[\mathrm{d}R]}{(2\pi)^N} \mathrm{e}^{-\frac{1}{2}\sum_{\alpha,\beta}(s^{-1})_{\alpha\beta}\,\mathrm{str}(R_\alpha R_\beta) + \mathrm{i}\sum_\alpha \mathrm{str}(R_\alpha X_\alpha X^\dagger_\alpha L^\pm)} , \quad (39)$$

where s denotes the symmetric matrix with $s_{\alpha\beta} = |\lambda^{(\tau)}_{\alpha\beta}|^2 \tilde{v}(E_\alpha - E_\beta)$ and s^{-1} is its inverse. Furthermore, the auxiliary (2×2) supermatrices R_α are parametrized as

$$R_\alpha := \begin{pmatrix} r_{1\alpha} & \rho_\alpha \\ \rho^*_\alpha & \mathrm{i} r_{2\alpha} \end{pmatrix} \tag{40}$$

with real-valued $r_{1\alpha}$, $r_{2\alpha}$ and Grassmann numbers ρ_α, ρ^*_α, and $[\mathrm{d}R] := \prod_\alpha \mathrm{d}R_\alpha$ with $\mathrm{d}R_\alpha := \mathrm{d}r_{1\alpha}\mathrm{d}r_{2\alpha}\mathrm{d}\rho_\alpha\mathrm{d}\rho^*_\alpha$ for short. Substituting Supplementary Eq. (39) into Supplementary Eq. (38) allows us to carry out the resulting Gaussian superintegral over X, leading to

$$\mathbb{E}[\mathcal{G}^{(\tau)}_{\mu\nu}(z^\pm)] = \delta_{\mu\nu} \int \frac{[\mathrm{d}R]}{(2\pi)^N} \left[(R_\mu + z^\pm - E_\mu)^{-1}\right]_{11} \mathrm{e}^{-\operatorname{str}\left[\frac{1}{2}\sum_{\alpha,\beta}(s^{-1})_{\alpha\beta} R_\alpha R_\beta + \sum_\alpha \ln(R_\alpha + z^\pm - E_\alpha)\right]} . \tag{41}$$

To calculate the remaining integral over the supermatrices R_α, we adopt a saddle-point approximation, exploiting that the exponent of the integrand in Supplementary Eq. (41) is extensive in $N \gg 1$ and thus dominated by the region around the highest saddle points of the exponent in the complex, multi-dimensional R plane along a suitably chosen integration contour. To find this stationary point, we look for supermatrices R_μ such that the first variation of the exponent in Supplementary Eq. (41) with respect to R vanishes, i.e.,

$$R_\mu + \sum_\alpha s_{\mu\alpha} (R_\alpha + z^\pm - E_\alpha)^{-1} = 0 . \tag{42}$$

From the possibly multiple solutions of this saddle-point equation, we have to select the dominant one that can be reached by a deformation of the original integration contour without crossing any singularities. The saddle-point approximation of Supplementary Eq. (41) is then obtained as the product of the integrand and the inverse square root of the superdeterminant corresponding to the second variation of the exponent in Supplementary Eq. (41), where both are evaluated at the dominating saddle point. Since the integrand is invariant under (pseudo)unitary transformations $R_\mu \mapsto T R_\mu T^{-1}$ with fixed T satisfying $T^\dagger L^\pm T = L^\pm$, further solutions can be generated from any given one $\hat{R}_\mu$ via $T\hat{R}_\mu T^{-1}$. Focusing on diagonal solutions first, the matrix-valued Supplementary Eq. (42) decouples into two identical equations for its entries. Consequently, any diagonal solution will be of the form $\hat{R}_\mu = \hat{r}(E_\mu, z^\pm, \tau)\mathbb{1}$ for some scalar function $\hat{r}(E_\mu, z^\pm, \tau)$ (explicitly indicating the dependence on E_μ, $z^\pm$, and τ again) such that

$$\hat{r}(E_\mu, z^\pm, \tau) + \sum_\alpha \frac{s_{\mu\alpha}}{z^\pm - E_\alpha + \hat{r}(E_\alpha, z^\pm, \tau)} = 0 . \tag{43}$$

Since $\hat{R}_\mu$ is proportional to the identity matrix, $T\hat{R}_\mu T^{-1} = \hat{R}_\mu$ and the equivalent solutions collapse back onto the diagonal one. Moreover, since the superdeterminant of any matrix proportional to $\mathbb{1}$ is unity, the contribution involving the second variation of the exponent in Supplementary Eq. (41) amounts to a trivial factor of one. After the saddle-point approximation, we thus find

$$\mathbb{E}[\mathcal{G}^{(\tau)}_{\mu\nu}(z^\pm)] = \frac{\delta_{\mu\nu}}{z^\pm - E_\mu + \hat{r}(E_\mu, z^\pm, \tau)} . \tag{44}$$

Finally, we rewrite the sum in Supplementary Eq. (43) as an integral over the density of states D_0 and exploit that the latter is approximately constant within the relevant energy window (see above Eq. (7) in the main paper). We also substitute $s_{\alpha\beta} = |\lambda^{(\tau)}_{\alpha\beta}|^2 \tilde{v}(E_\alpha - E_\beta)$ as defined below Supplementary Eq. (39). Adopting Supplementary Eq. (36) and Eq. (11) from the main paper, we can express it more explicitly as $s_{\alpha\beta} = [-a_\tau + (E_\alpha - E_\beta)^2 b_\tau]\tilde{v}(E_\alpha - E_\beta)$. This quantity is homogeneous in energy in that it only depends on the difference $E_\alpha - E_\beta$. Due to the homogeneous density of states, $\hat{r}(E_\mu, z^\pm, \tau)$ will thus only depend on the difference $z^\pm - E_\mu$ (and τ), too, i.e., $\hat{r}(E_\mu, z^\pm, \tau) = \hat{r}(z^\pm - E_\mu, \tau)$. Consequently, Supplementary Eq. (43) takes the form

$$\hat{r}(z^\pm - E, \tau) - \int \mathrm{d}E'\, D_0 \frac{a_\tau - (E - E')^2 b_\tau}{z^\pm - E' + \hat{r}(z^\pm - E', \tau)} = 0 \tag{45}$$

Finally, we define the function

$$G(z^\pm, \tau) := [z^\pm + \hat{r}(z^\pm, \tau)]^{-1} . \tag{46}$$

Substituting into Supplementary Eq. (43) and shifting the integration variable, we conclude that $G(z, \tau)$ satisfies Eq. (24) from the main paper. Moreover, we can exploit Supplementary Eqs. (32), (44), and (46) to confirm that

$$\begin{aligned} \mathbb{E}[\mathcal{G}^{(\tau)}_{\mu\nu}(z^\pm)] &= \mathbb{E}[{}_0\langle\mu|(z^\pm - H^{(\tau)})^{-1}|\nu\rangle_0] \\ &= \delta_{\mu\nu} G(z^\pm - E_\mu, \tau) \end{aligned} \tag{47}$$

as stated below Eq. (24) in the main paper. Furthermore, Supplementary Eqs. (27) and (33) imply that $u(E, \tau) = \lim_{\eta \to 0+} \operatorname{Im} G(E - \mathrm{i}\eta, \tau)/\pi D_0$. Together with Supplementary Eq. (29), this establishes Eq. (23) from the main paper.

Lastly, we exploit that the typical scale of $u(E, \tau)$ as a function of E is much larger than the level spacing. This can be roughly understood by observing that $u(E, \tau)$ quantifies how strongly eigenvectors of $H^{(\tau)}$ and

H_0 are mixed by the perturbation $V^{(\tau)}$, cf. Supplementary Eq. (27). Hence the energy scale of $u(E,\tau)$ is reciprocal to the response time scale of the system. Therefore, a natural response time necessitates that the energy scale is much larger than the exponentially small level spacing in macroscopic systems. A more detailed justification will be provided in Supplementary Note 8 (starting below Supplementary Eq. (57)). Crucially, this entails the same property for $\tilde{u}(E,\tau)$ from Supplementary Eq. (31) and thus implies that $\tilde{\rho}(\tau)$ from Supplementary Eq. (30) resembles a microcanonical density operator [49, 50]. Even if the initial state occupies only a few energy levels of the reference Hamiltonian, the perturbation $V^{(\tau)}$ typically spreads these populations out across a large number of neighboring states. Moreover, if the reference system satisfies the eigenstate thermalization hypothesis (ETH) [49, 51–53] then the expectation value $\langle A\rangle_{\bar{\rho}}$ in the diagonal ensemble $\bar{\rho} = \sum_\mu \rho_{\mu\mu}(0)|\mu\rangle_{00}\langle\mu|$ already coincides with the thermal equilibrium value A_{th} (see below Eq. (6) in the main paper), and this effect will only be reinforced by the additional averaging caused by the convolution with $\tilde{u}(E,\tau)$ in Supplementary Eq. (30). Hence we conclude that $\langle A\rangle_{\tilde{\rho}(\tau)} = A_{\mathrm{th}}$ for all practical purposes in Supplementary Eq. (28), which establishes Eq. (22) from the main paper.

Supplementary Note 7: Integro-differential representation of $\gamma_\tau(t)$

In this section, we derive the relation from Eq. (10) of the main paper for the response function $\gamma_\tau(t)$, which was defined in Supplementary Eq. (29) and was subsequently shown in Supplementary Note 6 [see remarks below Supplementary Eq. (47)] to be equivalent to Eq. (23) of the main paper.

Our starting point is this representation of $\gamma_\tau(t)$ from Eq. (23) of the main paper, which involves the ensemble-averaged resolvent $G(z,\tau)$ of $H^{(\tau)}$ defined in Supplementary Eq. (46). Below this equation, it was also established that the latter function $G(z,\tau)$ is the solution of the nonlinear integral equation (24) from the main paper. Writing $z = x - \mathrm{i}\eta$ with fixed $\eta > 0$, we multiply both sides of Eq. (24) in the main paper by $\mathrm{e}^{\mathrm{i}xt}$ and integrate over $x \in \mathbb{R}$. Defining

$$h_\eta(t,\tau) := \int \mathrm{d}x\, \mathrm{e}^{\mathrm{i}xt}\, G(x-\mathrm{i}\eta,\tau)\,, \tag{48}$$

this yields

$$-\mathrm{i}\left(\frac{\partial}{\partial t} + \eta\right) h_\eta(t,\tau) + \int \mathrm{d}E\, D_0\, \tilde{v}(E,\tau) \int \mathrm{d}x \int \mathrm{d}y\, \mathrm{e}^{-\mathrm{i}xt}\, G(x-\mathrm{i}\eta,\tau)\, G(y-\mathrm{i}\eta,\tau)\, \delta(x-y-E) = 2\pi\delta(t)\,, \tag{49}$$

where

$$\tilde{v}(E,\tau) := \left[a_\tau - E^2 b_\tau\right] \tilde{v}(E)\,. \tag{50}$$

By analogy with Eq. (8) in the main paper, we also introduce the corresponding Fourier transform

$$v(t,\tau) := \int \mathrm{d}E\, D_0\, \tilde{v}(E,\tau)\, \mathrm{e}^{\mathrm{i}Et}\,. \tag{51}$$

Next, we employ the Fourier identity $2\pi\delta(x) = \int \mathrm{d}s\, \mathrm{e}^{\mathrm{i}xs}$ to replace $\delta(x-y-E)$ on the left-hand side of Supplementary Eq. (49) by an integral. Exploiting the definitions from Supplementary Eqs. (51) and (48), we take the limit $\eta \to 0+$ and find

$$\frac{\partial h_{0+}(t,\tau)}{\partial t} - \int \frac{\mathrm{d}s}{2\pi\mathrm{i}}\, h_{0+}(t-s,\tau)\, h_{0+}(s,\tau)\, v(s,\tau) = 2\pi\mathrm{i}\,\delta(t)\,. \tag{52}$$

In view of the δ inhomogeneity on the right-hand side, we now make an ansatz of the form $h_{0+}(t,\tau) = 2\pi\mathrm{i}\,\Theta(t)\,\tilde{\gamma}_\tau(t)$, where $\Theta(t)$ is the Heaviside step function and $\tilde{\gamma}_\tau(t)$ is assumed to be a bounded and sufficiently smooth function of t. Substituting into Supplementary Eq. (52), we obtain

$$\Theta(t)\,\frac{\partial\tilde{\gamma}_\tau(t)}{\partial t} - \int_0^t \mathrm{d}s\, \tilde{\gamma}_\tau(t-s)\, \tilde{\gamma}_\tau(s)\, v(s,\tau) = 0\,. \tag{53}$$

Combining the relation (23) from the main paper and Supplementary Eq. (48), we find that $\gamma_\tau(t) = [h_{0+}(t,\tau) - h_{0+}(-t,\tau)^*]/2\pi\mathrm{i}$ and thus

$$\gamma_\tau(t) = \Theta(t)\,\tilde{\gamma}_\tau(t) + \Theta(-t)[\tilde{\gamma}_\tau(-t)]^*\,. \tag{54}$$

For $t > 0$, it follows that $\gamma_\tau(t)$ satisfies the integro-differential equation

$$\frac{\partial\gamma_\tau(t)}{\partial t} = \int_0^t \mathrm{d}s\, \gamma_\tau(t-s)\, \gamma_\tau(s)\, v(s,\tau)\,. \tag{55}$$

Likewise, for $t < 0$, Supplementary Eq. (55) follows by observing that $\tilde{v}(E,\tau)$ from Supplementary Eq. (50) is real-valued and even in E, implying the same for the Fourier transform $v(s,\tau)$ from Supplementary Eq. (51) as a function of s. Furthermore, the initial condition $\gamma_\tau(0) = 1$ as stated below Eq. (10) in the main paper and the bound $|\gamma_\tau(t)| \le 1$ are understood from Supplementary Eq. (29) and the definition of $u(E,\tau)$ from Supplementary Eq. (27) by observing that $\int \mathrm{d}E\, D_0\, u(E,\tau) \simeq \mathbb{E}[\sum_n |U^{(\tau)}_{n\mu}|^2] = 1$.

Finally, upon substitution of Supplementary Eq. (50) into (51) and partial integration, we find that

$$v(s,\tau) = \left[a_\tau + b_\tau \frac{\mathrm{d}^2}{\mathrm{d}s^2} \right] v(s)\,, \tag{56}$$

confirming that Supplementary Eq. (55) is equivalent to Eq. (10) from the main paper.

Supplementary Note 8: Ensemble variance and fluctuations of auxiliary dynamics

In this section, we establish the bound (25) from the main paper, which provides the concentration property that promotes the ensemble average from Eq. (22) of the main paper (see also Supplementary Note 6) to a prediction for nearly all individual perturbation operators in the ensemble, i.e., Eq. (26) of the main paper.

We first analyze the variance $\mathbb{E}[\xi(t,\tau)^2]$ of the deviations $\xi(t,\tau) = \langle A \rangle_{\rho(t,\tau)} - \mathbb{E}[\langle A \rangle_{\rho(t,\tau)}]$ between the dynamics for a particular perturbation and the ensemble-averaged behavior. From Supplementary Eq. (26), we understand that $\xi(t,\tau)^2$ involves eight factors of the basis transformation matrix elements $U^{(\tau)}_{n\mu} = \langle n(\tau) \mu \rangle_0$. Similarly as in Supplementary Note 6, the pertinent ensemble average can be broken down into contributions involving only the second-moment characteristic $u(E,\tau)$ from Supplementary Eq. (27) by adopting the methods of Ref. [43]. This yields the upper bound

$$\mathbb{E}[\xi(t,\tau)^2] \le c\,\Delta_A^2 \max_E u(E,\tau)\,, \tag{57}$$

where Δ_A is the measurement range of A (largest minus smallest eigenvalue) and c is a positive constant of order 10^3 or less, independent of any system details (particularly H_0, $f(t)$, $\tilde{v}(E)$, $\rho(0)$, or A).

To understand how this bound scales with the system size, we take a closer look at the function $u(E,\tau)$. According to its definition in Supplementary Eq. (27), $u(E,\tau)$ quantifies how much an eigenvector $|\mu\rangle_0$ of the reference Hamiltonian H_0 contributes to the eigenvector $|n(\tau)\rangle$ of $H^{(\tau)}$ that lies a distance $E = E_n - E_\mu$ away from it in the spectrum. Since the level density in generic many-body systems scales exponentially with the degrees of freedom N_{dof}, any driving operator V with a noticeable effect on the auxiliary Hamiltonian $H^{(\tau)}$ from Eq. (19) of the main paper will inevitably mix a large number N_v of energy levels [43], i.e.,

$$N_v = 10^{\mathcal{O}(N_{\mathrm{dof}})}\,. \tag{58}$$

Taking for granted that $u(E,\tau)$ is reasonably smooth, this implies that it will typically extend across a scale of order N_v/D_0 in E and that its maximum will be at most of order N_v^{-1}, i.e., $\max_E u(E,\tau) \lesssim N_v^{-1}$. Introducing a suitable constant

$$\delta \sim (c N_v^{-1})^{1/3} = 10^{-\mathcal{O}(N_{\mathrm{dof}})}\,, \tag{59}$$

we can thus rewrite Supplementary Eq. (57) as

$$\mathbb{E}[\xi(t,\tau)^2] \le \delta^3\,\Delta_A^2\,. \tag{60}$$

To reinforce Supplementary Eq. (58) further, we also note that perturbations mixing only a small number of energy levels will only induce changes of the dynamics on time scales of order D_0 because they only affect the corresponding frequencies $E_n^{(\tau)} - E_m^{(\tau)} \sim D_0^{-1}$ in Supplementary Eq. (26). Considering the extremely large level density, the time scale associated with D_0 is unimaginably large and typically exceeds the age of the universe by many orders of magnitude. For all times of practical interest, the dynamics under $H^{(\tau)}$ would thus be indistinguishable from the reference dynamics under H_0. Observing that $\gamma_\tau(t) \approx 1$ for these times according to Supplementary Eq. (29) and the normalization of $u(E,\tau)$, this limiting case is reflected correctly in Supplementary Eq. (28), meaning that perturbations violating Supplementary Eq. (58) are covered by the final result as well, despite being physically uninteresting.

Exploiting Chebyshev's inequality, we can bound the probability that the deviations $|\xi(t,\tau)|$ exceed a predefined threshold x in terms of the variance,

$$\mathbb{P}(|\xi(t,\tau)| \ge x) \le \frac{\mathbb{E}[\xi(t,\tau)^2]}{x^2}\,. \tag{61}$$

Utilizing Supplementary Eq. (60) and choosing $x = \delta \Delta_A$, we then obtain the relation (25) from the main paper.

[1] L. D'Alessio and A. Polkovnikov, Many-body energy localization transition in periodically driven systems, Ann. Phys. **333**, 19 (2013).

[2] L. D'Alessio and M. Rigol, Long-time behavior of isolated periodically driven interacting lattice systems, Phys. Rev. X **4**, 041048 (2014).

[3] A. Lazarides, A. Das, and R. Moessner, Equilibrium states of generic quantum systems subject to periodic driving, Phys. Rev. E **90**, 012110 (2014).

[4] P. Ponte, Z. Papić, F. Huveneers, and D. A. Abanin, Many-body localization in periodically driven systems, Phys. Rev. Lett. **114**, 140401 (2015).

[5] T. Ishii, T. Kuwahara, T. Mori, and N. Hatano, Heating in integrable time-periodic systems, Phys. Rev. Lett. **120**, 220602 (2018).

[6] K. Mallayya and M. Rigol, Heating rates in periodically driven strongly interacting quantum many-body systems, Phys. Rev. Lett. **123**, 240603 (2019).

[7] T. N. Ikeda and A. Polkovnikov, Fermi's golden rule for heating in strongly driven Floquet systems, Phys. Rev.

B **104**, 134308 (2021).
[8] T. Mori, Heating rates under fast periodic driving beyond linear response, Phys. Rev. Lett. **128**, 050604 (2022).
[9] A. Haldar, R. Moessner, and A. Das, Onset of Floquet thermalization, Phys. Rev. B **97**, 245122 (2018).
[10] D. A. Abanin, W. De Roeck, and F. Huveneers, Exponentially slow heating in periodically driven many-body systems, Phys. Rev. Lett. **115**, 256803 (2015).
[11] D. A. Abanin, W. De Roeck, W. W. Ho, and F. Huveneers, Effective Hamiltonians, prethermalization, and slow energy absorption in periodically driven many-body systems, Phys. Rev. B **95**, 014112 (2017).
[12] T. Mori, T. Kuwahara, and K. Saito, Rigorous bound on energy absorption and generic relaxation in periodically driven quantum systems, Phys. Rev. Lett. **116**, 120401 (2016).
[13] T. Kuwahara, T. Mori, and K. Saito, Floquet-Magnus theory and generic transient dynamics in periodically driven quantum systems, Ann. Phys. **367**, 96 (2016).
[14] D. V. Else, B. Bauer, and C. Nayak, Prethermal phases of matter protected by time-translation symmetry, Phys. Rev. X **7**, 011026 (2017).
[15] F. Machado, D. V. Else, G. D. Kahanamoku-Meyer, C. Nayak, and N. Y. Yao, Long-range prethermal phases of nonequilibrium matter, Phys. Rev. X **10**, 011043 (2020).
[16] A. Rubio-Abadal, M. Ippoliti, S. Hollerith, D. Wei, J. Rui, S. L. Sondhi, V. Khemani, C. Gross, and I. Bloch, Floquet prethermalization in a Bose-Hubbard System, Phys. Rev. X **10**, 021044 (2020).
[17] W. Beatrez, O. Janes, A. Akkiraju, A. Pillai, A. Oddo, P. Reshetikhin, E. Druga, M. McAllister, M. Elo, B. Gilbert, D. Suter, and A. Ajoy, Floquet prethermalization with lifetime exceeding 90 s in a bulk hyperpolarized solid, Phys. Rev. Lett. **127**, 170603 (2021).
[18] D. V. Else, B. Bauer, and C. Nayak, Floquet time crystals, Phys. Rev. Lett. **117**, 090402 (2016).
[19] N. Y. Yao, A. C. Potter, I.-D. Potirniche, and A. Vishwanath, Discrete time crystals: rigidity, criticality, and realizations, Phys. Rev. Lett. **118**, 030401 (2017).
[20] A. Russomanno, F. Iemini, M. Dalmonte, and R. Fazio, Floquet time crystal in the Lipkin-Meshkov-Glick model, Phys. Rev. B **95**, 214307 (2017).
[21] B. Zhu, J. Marino, N. Y. Yao, M. D. Lukin, and E. A. Demler, Dicke time crystals in driven-dissipative quantum many-body systems, New J. Phys. **21**, 073028 (2019).
[22] H. P. Ojeda Collado, G. Usaj, C. A. Balseiro, D. H. Zanette, and J. Lorenzana, Emergent parametric resonances and time-crystal phases in driven Bardeen-Cooper-Schrieffer systems, Phys. Rev. Research **3**, L042023 (2021).
[23] P. Kongkhambut, J. Skulte, L. Mathey, J. G. Cosme, A. Hemmerich, and H. Keßler, Observation of a continuous time crystal, Science **377**, 670 (2022).
[24] P. Hannaford and K. Sacha, A decade of time crystals: quo vadis?, EPL **139**, 10001 (2022).
[25] C. Bartsch and J. Gemmer, Dynamical typicality of quantum expectation values, Phys. Rev. Lett. **102**, 110403 (2009).
[26] R. Steinigeweg, J. Gemmer, and W. Brenig, Spin-current autocorrelations from single pure-state propagation, Phys. Rev. Lett. **112**, 120601 (2014).
[27] P. Reimann and J. Gemmer, Why are macroscopic experiments reproducible? Imitating the behavior of an ensemble by single pure states, Physica A **552**, 121840 (2020).
[28] L. Dabelow, P. Vorndamme, and P. Reimann, Modification of quantum many-body relaxation by perturbations exhibiting a banded matrix structure, Phys. Rev. Research **2**, 033210 (2020).
[29] A. Russomanno, A. Silva, and G. E. Santoro, Periodic steady regime and inference in a periodically driven quantum systems, Phys. Rev. Lett. **109**, 257201 (2012).
[30] A. Lazarides, A. Das, and R. Moessner, Periodic thermodynamics of isolated quantum systems, Phys. Rev. Lett. **112**, 150401 (2014).
[31] I. M. Georgescu, S. Ashhab, and F. Nori, Quantum simulation, Rev. Mod. Phys. **86**, 153 (2014).
[32] A. Lazarides, A. Das, and R. Moessner, Fate of many-body localization under periodic driving, Phys. Rev. Lett. **115**, 030402 (2015).
[33] M. Holthaus, Tutorial: Floquet engineering with quasienergy bands of periodically driven optical lattices, J. Phys. B **49**, 013001 (2016).
[34] R. Moessner and S. L. Sondhi, Equilibration and order in quantum Floquet matter, Nat. Phys. **13**, 424 (2017).
[35] D. A. Abanin, W. De Roeck, W. W. Ho, and F. Huveneers, A rigorous theory of many-body prethermalization for periodically driven and closed quantum systems, Commun. Math. Phys. **354**, 809 (2017).
[36] T. Oka and S. Kitamura, Floquet engineering of quantum materials, Annu. Rev. Cond. Mat. Phys. **10**, 387 (2019).
[37] P. Peng, C. Yin, X. Huang, C. Ramanathan, and P. Cappellaro, Floquet prethermalization in dipolar spin chains, Nat. Phys. **17**, 444 (2021).
[38] C. Weitenberg and J. Simonet, Tailoring quantum gases by Floquet engineering, Nat. Phys. **17**, 1342 (2021).
[39] S. Blanes, F. Casas, J. Oteo, and J. Ros, The Magnus expansion and some of its applications, Phys. Rep. **470**, 151 (2009).
[40] G. Teschl, *Ordinary Differential Equations and Dynamical Systems* (American Mathematical Society, Providence, Rhode Island, 2012).
[41] T. Langen, T. Gasenzer, and J. Schmiedmayer, Prethermalization and universal dynamics in near-integrable quantum systems, J. Stat. Mech. **2016**, 064009 (2016).
[42] T. Mori, T. N. Ikeda, E. Kaminishi, and M. Ueda, Thermalization and prethermalization in isolated quantum systems: a theoretical overview, J. Phys. B **51**, 112001 (2018).
[43] L. Dabelow and P. Reimann, Relaxation Theory for Perturbed Many-Body Quantum Systems versus Numerics and Experiment, Phys. Rev. Lett. **124**, 120602 (2020).
[44] A. D. Mirlin, Statistics of energy levels and eigenfunctions in disordered and chaotic systems: Supersymmetry approach, arXiv:cond-mat/9412060 (1996).
[45] F. Haake, *Quantum Signatures of Chaos* (Springer, Berlin, 2010).
[46] J. Verbaarschot, H. Weidenmüller, and M. Zirnbauer, Grassmann integration in stochastic quantum physics: The case of compound nucleus scattering, Phys. Rep. **129**, 367 (1985).
[47] K. Efetov, *Supersymmetry in Disorder and Chaos* (Cambridge University Press, Cambridge, UK, 1996).
[48] F. A. Berezin, *Introduction to Superanalysis* (Springer, Dordrecht, Netherlands, 2010).
[49] J. M. Deutsch, Quantum statistical mechanics in a closed system, Phys. Rev. A **43**, 2046 (1991).

[50] P. Reimann and L. Dabelow, Refining Deutsch's approach to thermalization, Phys. Rev. E **103**, 022119 (2021).
[51] M. Srednicki, Thermal fluctuations in quantized chaotic systems, J. Phys. A **29**, L75 (1996).
[52] M. Srednicki, The approach to thermal equilibrium in quantum chaotic systems, J. Phys. A **32**, 1163 (1999).
[53] M. Rigol, V. Dunjko, and M. Olshanii, Thermalization and its mechanism for generic isolated quantum systems, Nature **452**, 854 (2008).
